# Cohort profile: the Swiss Mother and Child HIV Cohort Study (MoCHiV)

**DOI:** 10.1136/bmjopen-2024-086543

**Published:** 2024-09-23

**Authors:** Paolo Paioni, Murezi Capaul, Anja Brunner, Anna Traytel, Karoline Aebi-Popp, Pierre-Alex Crisinel, Andrea Duppenthaler, Huldrych Günthard, Begona Martinez De Tejada, Lisa Kottanattu, Marcel Stöckle, Andri Rauch, Noemie Wagner, Irene Hösli, Christoph Rudin, Alexandra Scherrer, Katharina Kusejko, Christian R Kahlert, Swiss HIV Cohort Study (SHCS) and the Swiss Mother and Child HIV Cohort Study (MoCHiV), I Abela, K Aebi-Popp, A Anagnostopoulos, M Battegay, M Baumann, E Bernasconi, DL Braun, HC Bucher, A Calmy, M Cavassini, A Ciuffi, PA Crisinel, K Darling, A Duppenthaler, G Dollenmaier, M Egger, L Elzi, J Fehr, J Fellay, K Francini, H Furrer, CA Fux, HF Günthard, A Hachfeld, D Haerry, B Hasse, HH Hirsch, M Hoffmann, I Hösli, M Huber, D Jackson-Perry, CR Kahlert, L Kaiser, E Kapfhammer, O Keiser, T Klimkait, M Kohns, L Kottanattu, RD Kouyos, H Kovari, K Kusejko, N Labhardt, B Martinez de Tejada, C Marzolini, KJ Metzner, N Müller, J Nemeth, D Nicca, J Notter, P Paioni, G Pantaleo, M Perreau, Polli Ch, A Rauch, L Salazar-Vizcaya, P Schmid, R Speck, M Stöckle, P Tarr, M Thanh Lecompte, A Trkola, N Wagner, G Wandeler, M Weisser, S Yerly, J Böni, J-J Cheseaux, P Francioli, B Hirschel, C Kind, B Ledergerber, D Nadal, M Rickenbach, C Rudin, J Schüpbach, C-A Siegrist

**Affiliations:** 1 Division of Infectious Diseases and Hospital Epidemiology, University Children’s Hospital Zurich, Zurich, Switzerland; 2 Department of Infectious Diseases and Hospital Epidemiology, University Hospital Zurich, Zurich, Switzerland; 3 Frauenklinik, Bürgerspital Solothurn, Solothurn, Switzerland; 4 Institute of Medical Virology, University of Zurich, Zurich, Switzerland; 5 Department of Infectious Diseases, Bern University Hospital, University of Bern, Bern, Switzerland; 6 Department of Obstetrics and Gynecology, Lindenhofspital Bern, Bern, Switzerland; 7 Unit of Pediatric Infectious Diseases and Vaccinology, Department of Woman Mother and Child, Lausanne University Hospital and University of Lausanne, Lausanne, Switzerland; 8 Department of Pediatrics, Inselspital, Bern University Hospital, University of Bern, Bern, Switzerland; 9 Obstetrics Division. Department of Pediatrics, Gynecology and Obstetrics, University Hospitals Geneva, University of Geneva, Geneva, Switzerland; 10 Institute of Pediatrics of Southern Switzerland, EOC, Bellinzona, Switzerland; 11 Division of Infectious Diseases and Hospital Epidemiology, University Hospital Basel, University Basel, Basel, Switzerland; 12 Pediatric Infectious Diseases Unit, Department of Pediatrics, Gynecology and Obstetrics, Geneva University Hospitals, Geneve, Switzerland; 13 Department of Obstetrics, University Hospital Basel, Basel, Switzerland; 14 University Children's Hospital Basel, Basel, Switzerland; 15 Cantonal Hospital St. Gallen, Department of Infectious Diseases, Infection Prevention and Travel Medicine, St Gallen, Switzerland; 16 Children’s Hospital of Eastern Switzerland, Infectious Diseases and Hospital Epidemiology, St Gallen, Switzerland

**Keywords:** HIV & AIDS, Pregnant Women, Child

## Abstract

**Abstract:**

**Purpose:**

Prospective, multicentric observational cohort study in Switzerland investigating measures to prevent mother-to-child transmission in pregnant women with HIV (WWH) and assessing health and development of their exposed children as well as of children with HIV (CWH) in general.

**Participants:**

Between January 1986 and December 2022, a total of 1446 mother–child pairs were enrolled. During the same period, the study also registered 187 CWH and 521 HIV-exposed but uninfected children (HEU), for whom detailed maternal information was not available. Consequently, the cohort comprises a total of 2154 children.

**Findings to date:**

During these 37 years, research by the Swiss Mother and Child HIV Cohort Study (MoCHiV) and its international collaborators has strongly influenced the prevention of vertical transmission of HIV (eg, introduction and discontinuation of elective caesarean section, neonatal postexposure prophylaxis and breastfeeding). Contributions have also been made to the management of diagnostics (eg, p24 antigen assay) and the effects of antiretroviral treatment (eg, prematurity, growth) in HEU and CWH.

**Future plans:**

Most children present within the cohort are now HEU, highlighting the need to investigate other vertically transmitted pathogens such as hepatitis B and C viruses, cytomegalovirus or *Treponema pallidum*. In addition, analyses are planned on the longitudinal health status of CWH (eg, resistance and prolonged exposure to antiretroviral therapy), on social aspects including stigma in CWH and HEU, and on interventions to further optimise antenatal and postpartum care in WWH.

**STRENGTHS AND LIMITATIONS OF THIS STUDY**

- Prospective longitudinal data collection over more than 30 years and across generations, combining clinical, behavioural and laboratory data in conjunction with a biobank.
- Transfer of a large proportion of children with HIV in MoCHiV to the adult cohort allows vertically infected children to be followed well into adulthood.
- Standardised questionnaires, monitored data management and close cooperation with the Swiss HIV Cohort Study (SHCS) data centre guarantee high-quality longitudinal data.
- Fully standardised data are only available from 2004 as different databases have been used and had to be brought together as part of the development of MoCHiV and its integration into the SHCS and because various questionnaires were introduced at different times.
- Possible selection bias, as around 30% of pregnant women with HIV registered by the Swiss Federal Office of Public Health do not participate in the SHCS.

## Introduction

 The HIV pandemic continues to pose a significant challenge globally. According to the 2023 UNAIDS Fact Sheet, there are approximately 1.5 million children aged 0–14 years living with HIV, with around 130 000 new infections among children reported in 2022 alone. These data underscore the ongoing critical importance of addressing vertical transmission of HIV as a matter of global concern. While the highest prevalence of paediatric HIV infection is observed in sub-Saharan Africa, the global spread of the HIV pandemic, exacerbated in part by refugee movements, underscores the universal need for ongoing research into the prevention, diagnosis and treatment of HIV during pregnancy and among children affected. This still necessitates a concerted global effort to address HIV in all countries.

The Swiss Mother and Child HIV Cohort Study (MoCHiV) contributes as a nationwide ongoing prospective, multicentric, observational study following pregnant women living with HIV (WWH) and their children. In synergy with the Swiss HIV Cohort Study (SHCS, www.shcs.ch),^1 2^ its focus is on interdisciplinary clinical and translational research, epidemiological and social science and public health questions. MoCHiV provides access to a unique longitudinal data collection on WWH before, during and after pregnancy and delivery as well as on their offspring and on children with HIV (CWH). The primary objective is to evaluate and improve prevention against vertical transmission of HIV and secure optimal care of pregnant WWH and their exposed children, as well as of CWH. Epidemiological and psychosocial aspects are also examined, as are the multiple pharmacological exposures during pregnancy and childhood.

## Cohort description

### Setting, locations and relevant dates

**Figure 1:**
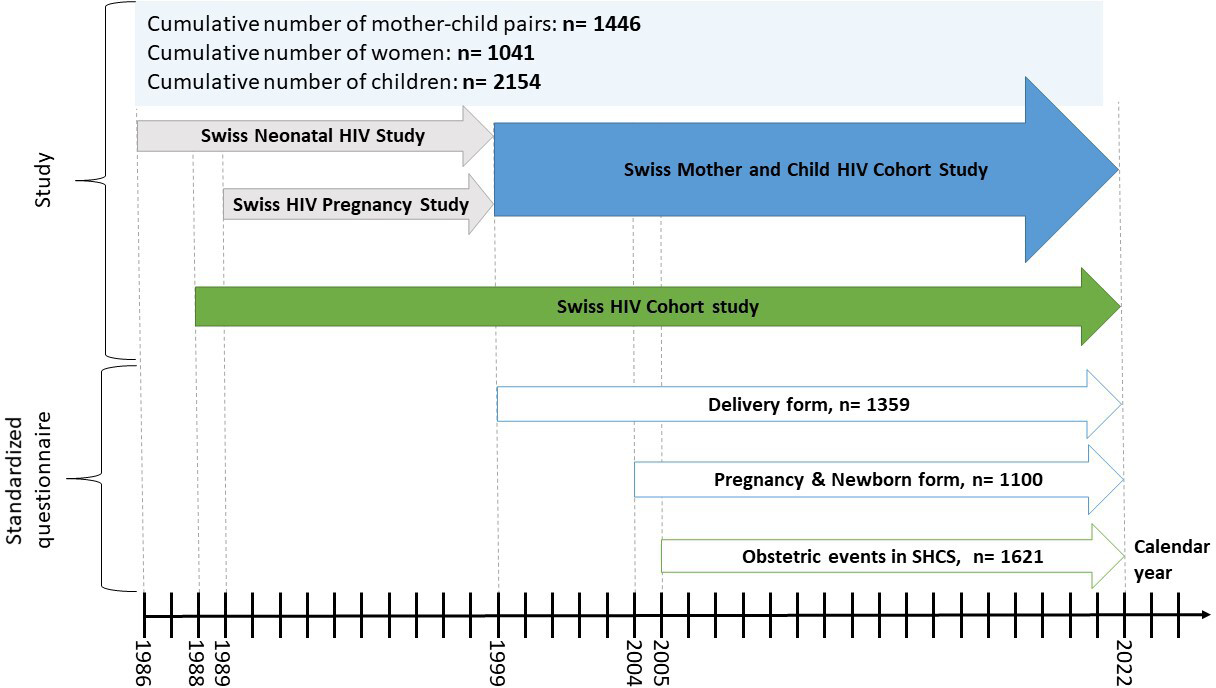
Overview of studies, numbers and introduction of standardised questionnaires over time. SHCS, Swiss HIV Cohort Study.

Two separate cohort studies existed in the 1980s: The Swiss Neonatal HIV Study,^3^ established in 1986, focused on paediatric HIV issues and the natural history of the infection in CWH. The Swiss HIV and Pregnancy Study,^4^ established in 1989, 1 year after the SHCS, focused on pregnant WWH and maternal or obstetric risk factors for vertical transmission. In 1999, these cohorts were combined to form a single mother and child cohort, named MoCHiV, which enrolled and followed pregnant WWH and their offspring (figure 1). In 2003, MoCHiV was integrated into the SHCS. Data are collected in seven centres (five Swiss university hospitals situated in Basel, Bern, Geneva, Lausanne and Zurich, and two cantonal reference hospitals situated in St. Gallen and Lugano), including private physicians and regional hospitals, which send their questionnaires to the nearest referral centre. Blood samples collected according to the study protocol are stored in a biobank at one of three central laboratories situated in Basel, Geneva and Zurich. The coordination and data centre in Zurich manages both cohort studies.

### Eligibility and follow-up

Participants eligible for inclusion in the cohort are (1) pregnant WWH who are registered in the SHCS, along with their children who are exposed to HIV and (2) CWH, regardless of whether their mothers are included in the SHCS. Subject to receipt of the signed consent forms, all pregnancies and children registered in the participating centres will be systematically included in this cohort. Pregnant WWH are followed semiannually according to the SHCS protocol and supplementary visits take place during pregnancy for antenatal care including monitoring of the HIV plasma viral load (pVL).

During the first 6 months of life, all children born to WWH have the same clinical follow-up. Diagnostic blood samples were initially taken at 1, 2 and 6 months of age. In recent years, due to the availability of more accurate HIV tests, only two samples at 1 and 6 months are taken to rule out vertical transmission of HIV by HIV RNA PCR. Additionally, at the age of 18–24 months, a negative antibody screening test is required. According to their HIV status, exposed children are followed according to separate follow-up programmes. CWH are examined every 6 months with in-depth information on laboratory and clinical parameters. HEU are followed every 6 months until 1 year of age, and then annually until 5 years of age (online supplemental figure 1) to assess growth and clinical events potentially related to combination antiretroviral therapy (cART) exposure in utero, through neonatal postexposure prophylaxis (nPEP) or breastfeeding.

CWH are offered to be transferred to the SHCS at the age of 18 years. There is no standardised transition programme, the process can vary between centres. However, a special questionnaire is used to collect information about participant’s drop-out, such as loss to follow-up or death. Clinical care is the same for all mothers and children, regardless of whether they are included in MoCHiV and/or the SHCS.

### Data collection

Until 2022, data have been prospectively collected via paper forms and submitted by mail to the data centre of the SHCS, where data managers transferred them manually to an Oracle database. During the second half of 2022, a user-friendly graphical user interface for electronic data collection at the local study sites was implemented and database migration to a Research Electronic Data Capture-based database (REDCap) was performed.^5^

Regarding the variables of interest, for pregnant women participating in the SHCS, we collected data on socioeconomic and educational background, general physical and mental health condition, relevant medical history, coinfections, comorbidities, specific HIV or AIDS-related diseases, current and past antiretroviral therapy, other medications, alcohol, nicotine or drug use as well as information about gynaecological and pregnancy history. Additionally, blood samples are regularly taken and stored in a biobank (plasma stored at −80°C and peripheral blood mononuclear cells (PBMCs) at – 160°C). During pregnancy, WWH participating in MoCHiV complete a structured pregnancy and delivery questionnaire, covering obstetric history, pregnancy complications and specific morbidity during each trimester of pregnancy and childbirth.

For children, the newborn questionnaire collects data on general health status, medication and feeding. Blood samples are taken at 1, 2 (not in all centres) and 6 months of age to rule out vertical transmission of HIV by HIV RNA PCR. Additionally, at the age of 18–24 months, a negative antibody screening test is required. The follow-up questionnaire includes anthropometry, social situation, health status, medication and nutrition. For CWH, information on HIV-associated diseases, treatment and laboratory results of haematological, biochemical and virological parameters is reported. Additionally, leftover routine blood samples are stored in a biobank as described above. For example, in 2022, 95 plasma samples and 73 PBMCs samples from children have been stored in our biobank.

### Consent and representativeness for Switzerland

Pregnant WWH participating in the SHCS sign an additional informed consent to participate in MoCHiV for themselves and simultaneously for their unborn child. All children born to WWH who signed the MoCHiV informed consent are enrolled, irrespective of their infection status. In addition, any CWH can participate, even if the mother is not known or not followed up in the SHCS.

SHCS and MoCHiV are highly representative of the group of pregnant WWH with an estimated coverage of 71% of all people with HIV (PWH) on cART in Switzerland.^2^ Overall, 86.1% (1041 of 1209) of the women giving birth enrolled in the SHCS agreed to participate in MoCHiV.

Tables1  2 present the cumulative numbers of the study participants and their characteristics. Online supplemental table 2 shows the cumulative numbers of mothers registered in the SHCS. Most reported characteristics are comparable between mothers participating in the SHCS only and mothers additionally participating in both SHCS and MoCHiV.

**Table 1:** Baseline characteristics of mothers and children registered in MoCHiV between January 1986 and December 2022

	Total	Zurich	Lausanne	Geneva	Basel	Bern	St. Gallen	Ticino
Mothers								
	1041	287	196	180	149	153	58	42
Total registered mothers								
Active patients	647 (62%)	150 (52%)	127 (65%)	115 (64%)	95 (64%)	107 (70%)	43 (74%)	24 (57%)
Voluntary Discontinuation	109 (10%)	28 (10%)	19 (10%)	24 (13%)	14 (9%)	15 (10%)	4 (7%)	8 (19%)
Died	55 (5%)	23 (8%)	3 (2%)	11 (6%)	7 (5%)	7 (5%)	4 (7%)	1 (2%)
Lost to follow-up	230 (22%)	86 (30%)	47 (24%)	30 (17%)	33 (22%)	24 (16%)	7 (12%)	9 (21%)
Age in years at HIV diagnosis								
Median (IQR)	26 (23–31)	26 (23–30)	27 (23–31)	26 (23–31)	26 (23–30)	27 (2–32)	27 (23–30)	27 (24–30)
Age in years at birth of the first child in SHCS								
Median (IQR)	30 (25–35)	30 (25–35)	31 (26–35)	29 (26–35)	30 (25–34)	29 (25–34)	30 (26–33)	32 (28–36)
Ethnicity								
Black	455 (44%)	86 (30%)	111 (57%)	96 (53%)	58 (39%)	86 (56%)	23 (40%)	4 (10%)
White	445 (43%)	164 (57%)	73 (37%)	63 (35%)	64 (43%)	47 (31%)	21 (36%)	26 (62%)
Asian	65 (6%)	19 (7%)	3 (2%)	4 (2%)	14 (9%)	13 (8%)	7 (12%)	6 (14%)
Hispano-American	37 (4%)	14 (5%)	5 (3%)	3 (2%)	5 (3%)	4 (3%)	2 (3%)	4 (10%)
Unknown	39 (4%)	4 (1%)	4 (2%)	14 (8%)	8 (5%)	3 (2%)	5 (9%)	2 (5%)
Source of HIV infection								
Heterosexual contact	818 (79%)	205 (71%)	165 (84%)	146 (81%)	115 (77%)	124 (81%)	44 (76%)	34 (81%)
Intravenous drug use	152 (15%)	66 (23%)	15 (8%)	18 (10%)	26 (17%)	14 (9%)	11 (19%)	8 (19%)
Perinatal transmission	18 (2%)	6 (2%)	5 (3%)	5 (3%)	1 (1%)	0 (0%)	1 (2%)	0 (0%)
Blood products	14 (1%)	3 (1%)	4 (2%)	4 (2%)	2 (1%)	1 (1%)	1 (2%)	0 (0%)
Unknown	39 (4%)	7 (2%)	7 (4%)	7 (4%)	5 (3%)	14 (9%)	1 (2%)	0 (0%)
Viral load at delivery (n=1446 for all deliveries to include mothers with multiple deliveries)								
Nr of deliveries	1446	388	266	258	201	203	76	54
<50 copies/mL	814 (56%)	211 (54%)	149 (56%)	145 (56%)	113 (56%)	128 (63%)	36 (47%)	32 (59%)
50–1000 copies/mL	132 (9%)	34 (9%)	29 (11%)	19 (7%)	14 (7%)	20 (10%)	9 (12%)	7 (13%)
>1000 copies/mL	79 (5%)	27 (7%)	17 (6%)	5 (2%)	18 (9%)	6 (3%)	4 (5%)	2 (4%)
Unknown	421 (29%)	116 (30%)	71 (27%)	89 (34%)	56 (28%)	49 (24%)	27 (36%)	13 (24%)
Children								
	2154	591	393	335	324	298	110	103
Total registered children								
Active patients	115 (5%)	33 (6%)	29 (7%)	26 (8%)	14 (4%)	4 (1%)	5 (5%)	4 (4%)
Transition to SHCS	113 (5%)	29 (5%)	17 (4%)	21 (6%)	18 (6%)	18 (6%)	8 (7%)	2 (2%)
Completed follow-up	1247 (58%)	373 (63%)	245 (62%)	167 (50%)	189 (58%)	139 (47%)	63 (57%)	71 (69%)
Voluntary discontinuation	101 (5%)	49 (8%)	13 (3%)	16 (5%)	7 (2%)	10 (3%)	2 (2%)	4 (4%)
Died*	81 (4%)	31 (5%)	14 (4%)	11 (3%)	6 (2%)	9 (3%)	2 (2%)	8 (8%)
Lost to follow-up	497 (23%)	76 (13%)	75 (19%)	94 (28%)	90 (28%)	118 (40%)	30 (27%)	14 (14%)
Infection status								
Exposed uninfected	1732 (80%)	488 (83%)	331 (84%)	246 (73%)	249 (77%)	248 (83%)	88 (80%)	82 (80%)
Infected	285 (13%)	81 (14%)	47 (12%)	52 (16%)	37 (11%)	37 (12%)	16 (15%)	15 (15%)
Unknown	137 (6%)	22 (4%)	15 (4%)	37 (11%)	38 (12%)	13 (4%)	6 (5%)	6 (6%)
Sex								
Male	1099 (51%)	315 (53%)	191 (49%)	161 (48%)	161 (50%)	161 (54%)	57 (52%)	53 (51%)
Female	1055 (49%)	276 (47%)	202 (51%)	174 (52%)	163 (50%)	137 (46%)	53 (48%)	50 (49%)
Mode of delivery								
Caesarean	916 (43%)	309 (52%)	140 (36%)	131 (39%)	119 (37%)	127 (43%)	47 (43%)	43 (42%)
Vaginal	383 (18%)	56 (9%)	74 (19%)	105 (31%)	64 (20%)	58 (19%)	23 (21%)	3 (3%)
Unknown	855 (40%)	226 (38%)	179 (46%)	99 (30%)	141 (44%)	113 (38%)	40 (36%)	57 (55%)
Preterm birth								
Full-term birth	1652 (77%)	421 (71%)	297 (76%)	270 (81%)	258 (80%)	248 (83%)	78 (71%)	80 (78%)
Preterm birth	328 (15%)	119 (20%)	54 (14%)	43 (13%)	46 (14%)	29 (10%)	21 (19%)	16 (16%)
Unknown	174 (8%)	51 (9%)	42 (11%)	22 (7%)	20 (6%)	21 (7%)	11 (10%)	7 (7%)

* Cause of death overall: HIV (n=53), SIDS (n=9), child abuse (n=3), others (n=16).

CWHchildren with HIVMoCHiVSwiss Mother and Child HIV Cohort StudySHCSSwiss HIV Cohort StudySIDSsudden infant death syndrome

**Table 2:** Baseline characteristics of CWH registered in MoCHiV between January 1986 and December 2022

	Total	Zurich	Lausanne	Geneva	Basel	Bern	St. Gallen	Ticino
CWH included in MoCHiV								
	285	81	47	52	37	37	16	15
Total infected children								
Active patients	16 (6%)	9 (11%)	5 (11%)	0 (0%)	0 (0%)	0 (0%)	0 (0%)	2 (13%)
Transition to SHCS	113 (40%)	29 (36%)	17 (36%)	21 (40%)	18 (49%)	18 (49%)	8 (50%)	2 (13%)
Voluntary discontinuation	15 (5%)	6 (7%)	2 (4%)	4 (8%)	2 (5%)	1 (3%)	0 (0%)	0 (0%)
Died*	61 (21%)	23 (28%)	10 (21%)	7 (13%)	6 (16%)	5 (14%)	2 (12%)	8 (53%)
Lost to follow-up	80 (28%)	14 (17%)	13 (28%)	20 (38%)	11 (30%)	13 (35%)	6 (38%)	3 (20%)
Participation of mother in SHCS								
No participation	187 (66%)	56 (69%)	32 (68%)	31 (60%)	27 (73%)	23 (62%)	6 (38%)	12 (80%)
Participation	98 (34%)	25 (31%)	15 (32%)	21 (40%)	10 (27%)	14 (38%)	10 (62%)	3 (20%)
Age of inclusion								
At birth	75 (26%)	20 (25%)	11 (23%)	15 (29%)	12 (32%)	4 (11%)	4 (25%)	9 (60%)
Later	210 (74%)	61 (75%)	36 (77%)	37 (71%)	25 (68%)	33 (89%)	12 (75%)	6 (40%)
Median age at inclusion if later (IQR)	3.8 (1.2–8.2)	2.5 (0.9–5.1)	5.6 (2.1–10.6)	5.9 (3.5–10.6)	4 (0.9–10)	3.4 (1.6–6.3)	4.2 (1.2–6.3)	2 (0.8–3)
Sex								
Female	150 (53%)	42 (52%)	25 (53%)	29 (56%)	21 (57%)	16 (43%)	7 (44%)	10 (67%)
Male	135 (47%)	39 (48%)	22 (47%)	23 (44%)	16 (43%)	21 (57%)	9 (56%)	5 (33%)
Ethnicity								
White	82 (29%)	28 (35%)	15 (32%)	11 (21%)	12 (32%)	13 (35%)	2 (12%)	1 (7%)
Black	74 (26%)	9 (11%)	14 (30%)	21 (40%)	15 (41%)	7 (19%)	6 (38%)	2 (13%)
Hispano-American	13 (5%)	2 (2%)	3 (6%)	3 (6%)	0 (0%)	2 (5%)	3 (19%)	0 (0%)
Asian	8 (3%)	2 (2%)	0 (0%)	2 (4%)	1 (3%)	2 (5%)	1 (6%)	0 (0%)
Unknown	108 (38%)	40 (49%)	15 (32%)	15 (29%)	9 (24%)	13 (35%)	4 (25%)	12 (80%)
Source of HIV infection								
Vertical transmission	217 (76%)	59 (73%)	37 (79%)	47 (90%)	24 (65%)	29 (78%)	12 (75%)	9 (60%)
Blood products	4 (1%)	1 (1%)	2 (4%)	1 (2%)	0 (0%)	0 (0%)	0 (0%)	0 (0%)
Heterosexual contact	1 (0%)	0 (0%)	0 (0%)	0 (0%)	1 (3%)	0 (0%)	0 (0%)	0 (0%)
Unknown	63 (22%)	21 (26%)	8 (17%)	4 (8%)	12 (32%)	8 (22%)	4 (25%)	6 (40%)
Median follow-up time in years								
Median (IQR)	8.3 (2.0–15)	7.9 (1.5–16)	6.9 (2.2–12)	9.2 (2.6–15)	9.4 (3.1–16)	11 (5.8–15)	4.1 (0–13)	1.3 (0–10)

* Cause of death overall: HIV (n=53), SIDS (n=2), others (n=6).

CWHchildren with HIVMoCHiVSwiss Mother and Child HIV Cohort StudySHCSSwiss HIV Cohort StudySIDSsudden infant death syndrome

### Numbers of pregnant WWH and vertical transmission

Between January 1986 and December 2022, a total of 1041 pregnant WWH were registered in MoCHiV. Median age of women at birth of their first child was 30 years (IQR 25–35) and median age at HIV diagnosis was 26 years (IQR 23–31). 27.7% (288 of 1041) of the WWH were first diagnosed during pregnancy. The most common mode of HIV infection was heterosexual transmission in 78.6% of the women (818 of 1041), followed by intravenous drug use in 14.6% (152 of 1041) and vertical transmission in 1.7% of the cases (18 of 1041). Ethnicities are represented as follows: 43.7% (455 of 1041) black, 42.7% (445 of 1041) white, 6.2% (65 of 1041) Asian and 3.6% (37 of 1041) Hispano-American. There were 15% (328 of 2154) preterm deliveries (<37+0 weeks of gestation), of which 71% (233 of 328) were late preterm births (between 34+0 and 36+6 weeks of gestation).

A total of 1446 children were born from the 1041 WWH mentioned above, with 335 mothers having more than one child registered (280 mothers having 2, 42 mothers having 3, 11 mothers having 4 and 2 mothers having 5 children registered). In total, 25 twins and 1 triplet were born. Overall, 98 children turned out to be diagnosed with HIV resulting in an overall vertical transmission frequency of 6.8% (98 of 1446) spanning 37 years.

### Numbers of HIV-exposed children and CWH

Between January 1986 and December 2022, cumulative data on 2154 children were registered in MoCHiV, of whom 285 were CWH and 137 had an unknown infection status. In addition to the 1446 children mentioned above (1348 HIV-exposed children, 98 CWH), data of 708 (521 HIV-exposed children and 187 CWH) children without detailed information about the mother have been collected. On the other hand, a total of 285 CWH were enrolled, of which 95 (of 285, 33.3 %) were lost to follow-up or voluntarily withdrew from the study (eg, changed to a physician not participating in the SHCS), 61 (of 285, 21.4 %) died and 16 (of 285, 5.6%) are currently still being followed. 113 of 285 (39.6 %) children have reached adulthood and are now followed within the SHCS.^2^ The median age at the last visit to MoCHiV before transfer to the SHCS was 18.0 years (IQR 16.5–18.8).

### Findings to date

Vertical transmission of HIV has been significantly reduced to less than 1% in high-income countries over the past 30 years. This has been achieved through the introduction of various prevention measures, most importantly effective maternal cART. This was accompanied by corresponding changes in guidelines and standards of care, such as the change in recommended mode of delivery to elective caesarean section (eCS) or, later, the introduction of effective cART during pregnancy. Research from MoCHiV and its international collaborators has strongly influenced the recommendations of prevention measures over the past decades. MoCHiV is collaborating among others with the European Collaborative Study (ECS), the PENTA Foundation, the HIV Paediatric Prognostic Markers Collaborative Study (HPPMCS), the European Pregnancy and Paediatric Infections Cohort Collaboration (EPPICC) and the Collaborative Initiative for Paediatric HIV Education and Research (CIPHER).

In 1994, the Pediatric AIDS Clinical Trials Group Protocol (PACTG-076) Study Group published the first important general results on the reduction of vertical transmission of HIV. Zidovudine given orally during pregnancy, intravenously during delivery and orally to the newborn for 6 weeks reduced vertical transmission by two-thirds to 8% (PACTG-076).^6^ Based on these findings the Swiss Pediatric AIDS-Group recommended to offer zidovudine chemoprophylaxis to all pregnant WWH and their neonates in Switzerland in November 1994.^7^

Subsequently, the protective effect of eCS, before rupture of membranes and onset of labour has been demonstrated by Kind *et al* using data from the Swiss Neonatal HIV Study.^8 9^ In 1998, the same study group presented their results on the cumulative protective effect of both the eCS and the zidovudine chemoprophylaxis.^10^ Two international collaborative studies including data from the former two MoCHiV studies (the Swiss HIV and Pregnancy Study and the Swiss Neonatal HIV Study) confirmed these results in 1999.^11 12^

In the mid-1990s, new antiretroviral agents and combined treatment were introduced into clinical care. By combining three drugs including a protease inhibitor, antiretroviral treatment for the first time achieved complete suppression of HIV pVL in most patients. These new treatments and strategies were rapidly introduced into the SHCS with remarkable success at the population level: in 1999 90.7% of SHCS participants achieved an undetectable pVL (cut-off at that time<400 HIV-RNA copies/mL) after 12 months of treatment.^13 14^

**Figure 2:**
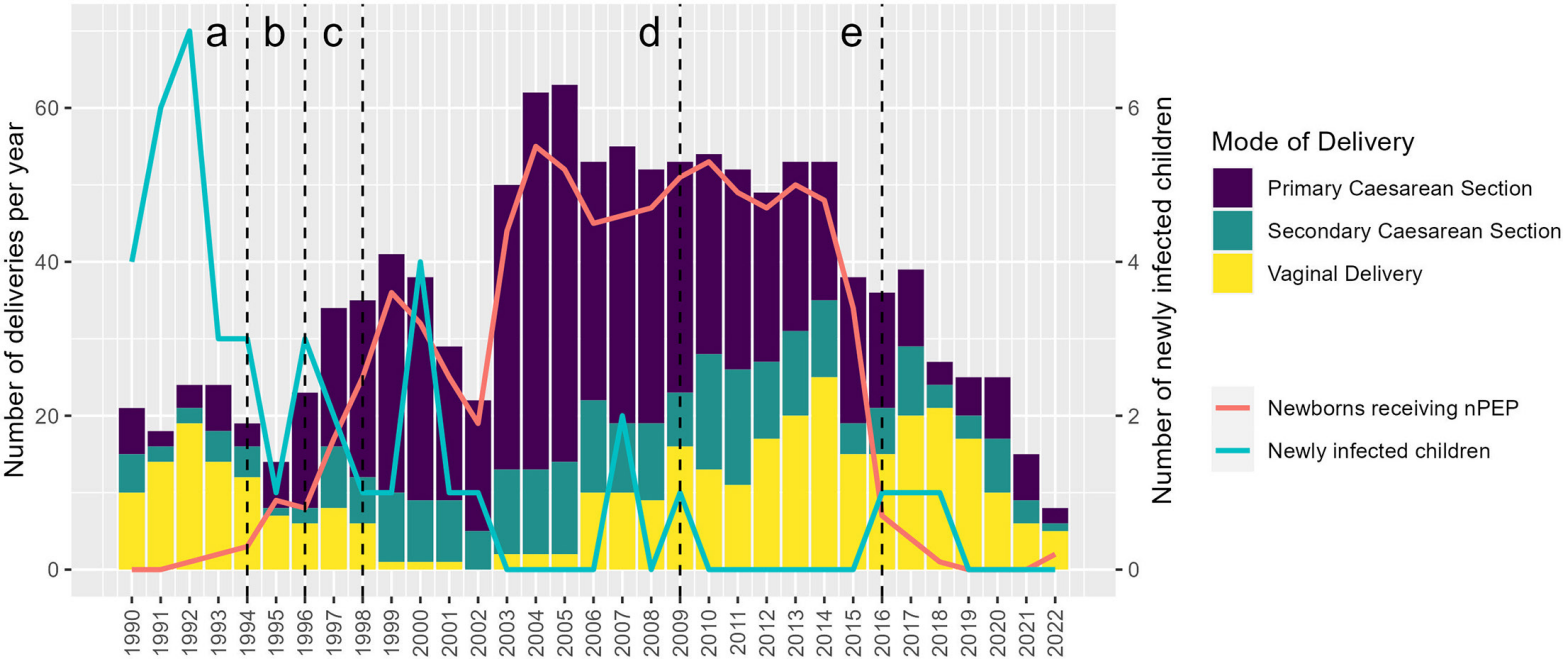
Number of newly infected children, children receiving neonatal postexposure prophylaxis (nPEP) and mode of delivery over time.

Initial concerns about the adverse effects of antiretrovirals on the unborn child have been refuted in various studies, among others by Lorenzi *et al* with data from the Swiss Neonatal HIV Study and the Swiss HIV and Pregnancy Study.^15^ In 1998, the Swiss guidelines were adapted and cART became the standard of care for pregnant WWH.^16^ This change in treatment regimen reduced HIV pVL to below the detection limit in the majority of WWH. As a result, the general recommendation of eCS for all pregnant WWH has been questioned in the light of outcome studies showing increased complication rates compared with vaginal delivery. In 2006, data from MoCHiV demonstrated that minor complications were eightfold more common in WWH undergoing an eCS than in WWH with vaginal delivery and this was independent of CD4 cell count or HIV pVL at the time of delivery.^17^ Accordingly, in 2009, Switzerland changed its guidelines and recommended vaginal delivery for women under cART during pregnancy and with undetectable HIV pVL near the time of delivery. Under these conditions, pregnant WWH can be managed in the same way as HIV-uninfected women.^18 19^ The corresponding decrease in eCS after 2009 is shown in figure 2. Also based on MoCHiV data, the association between cART and a higher rate of premature delivery was first reported by Lorenzi *et al*.^15^ In the following years, a study from Switzerland based on MoCHiV data and a collaborative study of three observational studies from the USA, the UK and the European Collaborative Study confirmed that cART during pregnancy was indeed associated with an increased risk of late preterm delivery compared with no therapy or antiretroviral monotherapy.^20 21^ The most recent analysis of all registered pregnancies in MoCHiV showed that the rate of preterm delivery in WWH has decreased in Switzerland between 1986 and 2020, but remains twice as high as in the general population.^22^

New, more effective agents should, therefore, always be assessed for safety in exposed children. In a collaborative project with the Pediatric HIV/AIDS Cohort Study in the USA, dolutegravir-based cART was shown to be safe and effective during pregnancy.^23^

By following HIV-exposed uninfected children (HEU) up to the age of 5 years, MoCHiV contributed to assess the short and long-term effects of intrauterine exposure to cART during pregnancy.^15 20 24^ A single-centre evaluation from Zürich showed that HEU receiving nPEP with zidovudine had nonsignificant trends of lower CD4 and CD8 T cells and CD19 B-cells than those who did not. This suggests toxicity that could affect the overall health of these children.^25^

The improvement of diagnostic procedures was another important research field of MoCHiV. As one of the first cohorts, the Swiss Neonatal HIV Study described repeated PCR to recognise perinatal HIV infection.^26^ Another important achievement was the development of heat-denatured p24 antigen assay by Schüpbach *et al*, using material collected by the Swiss Neonatal HIV study.^27^,^30^

In addition to eCS and maternal antiretroviral therapy, other measures to prevent vertical transmission of HIV include nPEP and formula feeding. These measures have also been questioned in the light of highly effective maternal antiretroviral therapy. In 2016, Switzerland became the first country worldwide to recommend discontinuation of the nPEP in newborns born to WWH with fully suppressed HIV pVL at the time of delivery, irrespective of the mode of delivery. This recommendation was issued by a working group of MoCHiV members together with the Federal Office of Public Health (FOPH).^31^ The implementation of the new recommendation was presented at the 17th European AIDS Conference (EACS 2019) in Basel (Abstract PS1/5).^32^ The rapidly decreasing number of newborns receiving nPEP after 2016 is shown in figure 2.

In 2018, new guidelines on breastfeeding in WWH were published. This made Switzerland the first country in a resource-rich setting to no longer rigidly discourage breastfeeding in WWH, provided certain defined conditions are met and monitoring is ensured.^33^ These conditions are referred to as the ‘optimal scenario’ and include a fully suppressed maternal HIV viral load throughout pregnancy. If the optimal scenario conditions are met, breastfeeding is supported if the mother wishes to breastfeed. However, the decision is preceded by a shared decision-making process to ensure that the pregnant woman fully understands the risks and benefits of breastfeeding in HIV. Thus, there is currently no recommendation for breastfeeding with HIV in Switzerland. The implementation of the new approach was analysed in a study describing the main motivational factors of WWH enrolled in MoCHiV who decided to breastfeed after a shared decision-making process.^34^ At the same time, the transfer of antiretroviral drugs into breast milk and the corresponding drug exposure of the infant were measured in the same patients.^35^ Additionally, retention in HIV care and viral suppression during the critical postpartum period has been assessed in MoCHiV overall^36^ and for the population of WWH with undetectable HIV pVL around the time of delivery,^37^ showing reassuringly high rates of both in the group of WWH with undetectable HIV pVL.

Based on the findings of the MoCHiV Cohort Study in the context of global research, the recommendations in Switzerland have constantly been adapted over the past decades. Figure 2 illustrates the changes in the Swiss recommendations, that is, the introduction and discontinuation of prevention measures against and a significant reduction in vertical transmission of HIV. This is illustrated by the decreasing number of newly infected children born to mothers registered in the SHCS database over time.

By following CWH up to the age of 18, the MoCHiV Study was involved in the investigation of HIV disease characteristics and therapy in children. Rudin *et al* evaluated the long-term safety and effectiveness of PI-based treatment regimes in CWH.^38 39^ MoCHiV early described catch-up growth in CWH after initiation of antiretroviral treatment with highly active antiretroviral therapy including protease inhibitors.^40^,^42^ The unique setting of MoCHiV with its direct link to the adult cohort allowed Compagno *et al* to assess the vertical transmission of antiretroviral drug-resistant HIV strains in CWH.^43^

### Strengths and limitations

**Figure 3:**
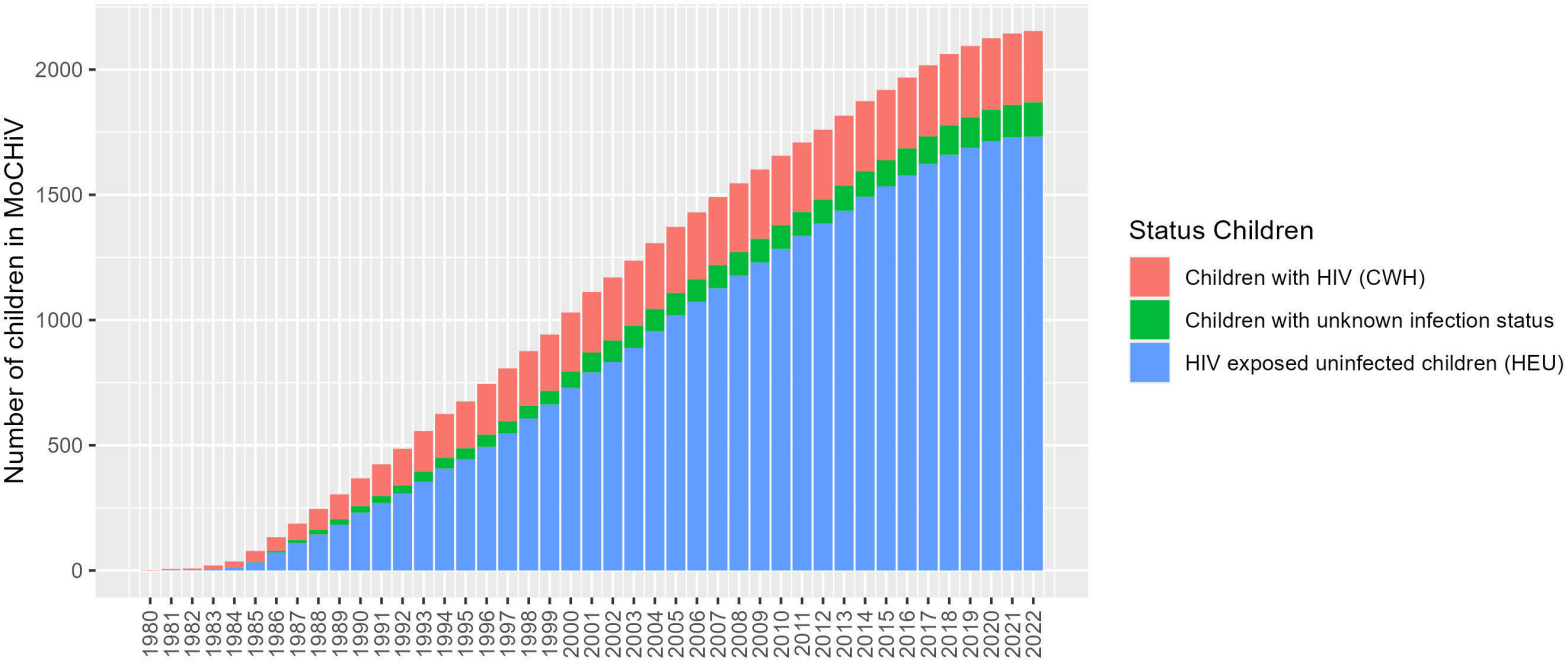
Cumulative number of children registered in the Mother and Child HIV Cohort Study.

The uniqueness of our cohort study is the prospective longitudinal data collection over more than 30 years and across generations, combining clinical, behavioural and laboratory data in conjunction with a biobank and a profound and long-standing synergy with the SHCS. Figure 3 shows the cumulative number of included children over the years.

Data from WWH are available in the SHCS from before and after pregnancy and in MoCHiV for the time during pregnancy and delivery. The link to the SHCS database provides detailed information on longitudinal maternal disease characteristics, including treatment and drug resistance, as well as epidemiological and socioeconomic data. The transfer of a large proportion of CWH in MoCHiV to the adult cohort also allows vertically infected children to be followed well into adulthood.

The inclusion of HEU is an additional strength. These children are followed for up to 5 years, enabling research into the long-term effects and adverse effects of antiretroviral exposure during pregnancy, delivery and the postnatal period.^25^ Additionally, stored plasma/serum, viable cells/cell pellets and placental material are available for analysis.

The standardised questionnaires monitored data management and close cooperation with the SHCS data centre guarantee high-quality longitudinal data. This data quality enables MoCHiV not only to answer its own research questions but also to participate actively in European and international collaborations.

Another major strength of the study is the interdisciplinary collaboration. Infectious disease specialists, gynaecologists, paediatricians and epidemiologists are represented on the MoCHiV board. This ensures that MoCHiV procedures and questionnaires are constantly adapted to the current guidelines and standards of care.

Some limitations of this study should be noted. There is no control group of pregnant women and their CWH. There may be a selection bias, as around 30% of pregnant WWH registered by the FOPH in Switzerland do not participate in the SHCS.^44^

Also, the number of patients per year is small and complete data collection also depends on the participating physicians and the completeness of the questionnaires.

Since different databases have been used and had to be brought together as part of the development of MoCHiV and its integration into the SHCS and because various questionnaires were introduced at different times as shown in figure 1, fully standardised data are only available after 2004. This makes the analysis of cumulative data, including data prior to 2004, somewhat difficult and challenging. The relatively high number of patients lost to follow-up as shown in table 1 is an additional difficulty that complicates longitudinal analysis.

### Collaboration

As described in the findings to date and strengths of the cohort, collaboration with other cohorts such as the EPPICC is already in place and is considered important. In principle, any access to cohort data or samples to answer a research question, whether internal or external, or the request for collaboration requires the approval of the scientific board. For this purpose, a structured and detailed study proposal must be submitted. Biological samples were used, for example, to assess the rate of vertical transmission of antiretroviral drug-resistant HIV strains in CWH.^43^ Further information regarding the cohort biobank can be found in previous publications^1 2^ The template, submission deadlines and a description of the evaluation and decision-making process can be found on the website (http://www.shcs.ch/132-who-can-submit).

### Patient and public involvement

Patient representatives have been on the scientific board for several years, ensuring active patient involvement in research. News and press releases are available on the cohort website and can be subscribed to as a newsletter.

### Study participation and study information

Physicians and regional hospital centers interested in a collaboration can contact one of the local centers for more information. Patients interested in participating can contact a collaborating physician, a regional hospital or directly one of the seven outpatient clinics. The SHCS has a website covering the most important information, including all questionnaires for MoCHiV and SHCS: http://www.shcs.ch.

## supplementary material

10.1136/bmjopen-2024-086543 online supplemental file 1

10.1136/bmjopen-2024-086543 online supplemental file 2

## Data Availability

Data are available on reasonable request.
