# Supplementary material for: Cohort profile: the Swiss Mother and Child HIV Cohort Study (MoCHiV)
Source: BMJ Open. 2024 Sep 23;14(9):e086543. doi: 10.1136/bmjopen-2024-086543 (PMC11418562; doi:10.1136/bmjopen-2024-086543)
Supplement: online supplemental file 1 [file bmjopen-14-9-s001.pdf]

Supplementary Table 1 Baseline characteristics of women giving birth in SHCS and MoCHiV between 01/1986 and 12/2022

	Total	Registered in MoCHiV	Not registered in MoCHiV	*p-value*[a]
**Mothers**				
	1209	1041	168	
**Total mothers registered in SHCS**				**< 0.001**
Active patients	777 (64 %)	647 (62 %)	130 (77 %)	
Voluntary	115 (10 %)	109 (10 %)	6 (4 %)	
Died	59 (5 %)	55 (5 %)	4 (2 %)	
Lost to follow-up	258 (21 %)	230 (22 %)	28 (17 %)	
**Age in years at HIV diagnosis**				**0.015**
Median (IQR)	27 [23, 31]	26 [23, 31]	28 [23, 32]	
**Ethnicity**				**0.047**
Black	540 (45 %)	455 (44 %)	85 (51 %)	
White	513 (42 %)	445 (43 %)	68 (40 %)	
Asian	72 (6 %)	65 (6 %)	7 (4 %)	
Hispano-American	45 (4 %)	37 (4 %)	8 (5 %)	
Unknown	39 (3 %)	39 (4 %)	0 (0 %)	
**Source of HIV infection**				**0.003**
Heterosexual Contact	951 (79 %)	818 (79 %)	133 (79 %)	
Intravenous drug use	164 (14 %)	152 (15 %)	12 (7 %)	
Perinatal transmission	22 (2 %)	18 (2 %)	4 (2 %)	
Blood Products	19 (2 %)	14 (1 %)	5 (3 %)	
Unknown	53 (4 %)	39 (4 %)	14 (8 %)	
**Viral load at delivery**				**< 0.001**
< 50 copies/ml	663 (55 %)	577 (55 %)	86 (51 %)	
50- 1000 copies/ml	116 (10 %)	106 (10 %)	10 (6 %)	
> 1000 copies/ml	119 (10 %)	114 (11 %)	5 (3 %)	
Log10 of median if >	4 [3.5, 4.4]	4 [3.5, 4.4]	4.1 [3.5, 4.3]	
Unknown	311 (26 %)	244 (23 %)	67 (40 %)	

[a] Chi-Square test for categorical variables and Welch’s t-test for continuous variable were used

Abbreviations: **IQR**, interquartile range; **MoCHiV**, Swiss Mother and Child HIV Cohort Study; **SHCS**, Swiss HIV Cohort Study
