# Supplementary figures and images for: Cohort profile: the Swiss Mother and Child HIV Cohort Study (MoCHiV)
Source: BMJ Open. 2024 Sep 23;14(9):e086543. doi: 10.1136/bmjopen-2024-086543 (PMC11418562; doi:10.1136/bmjopen-2024-086543)

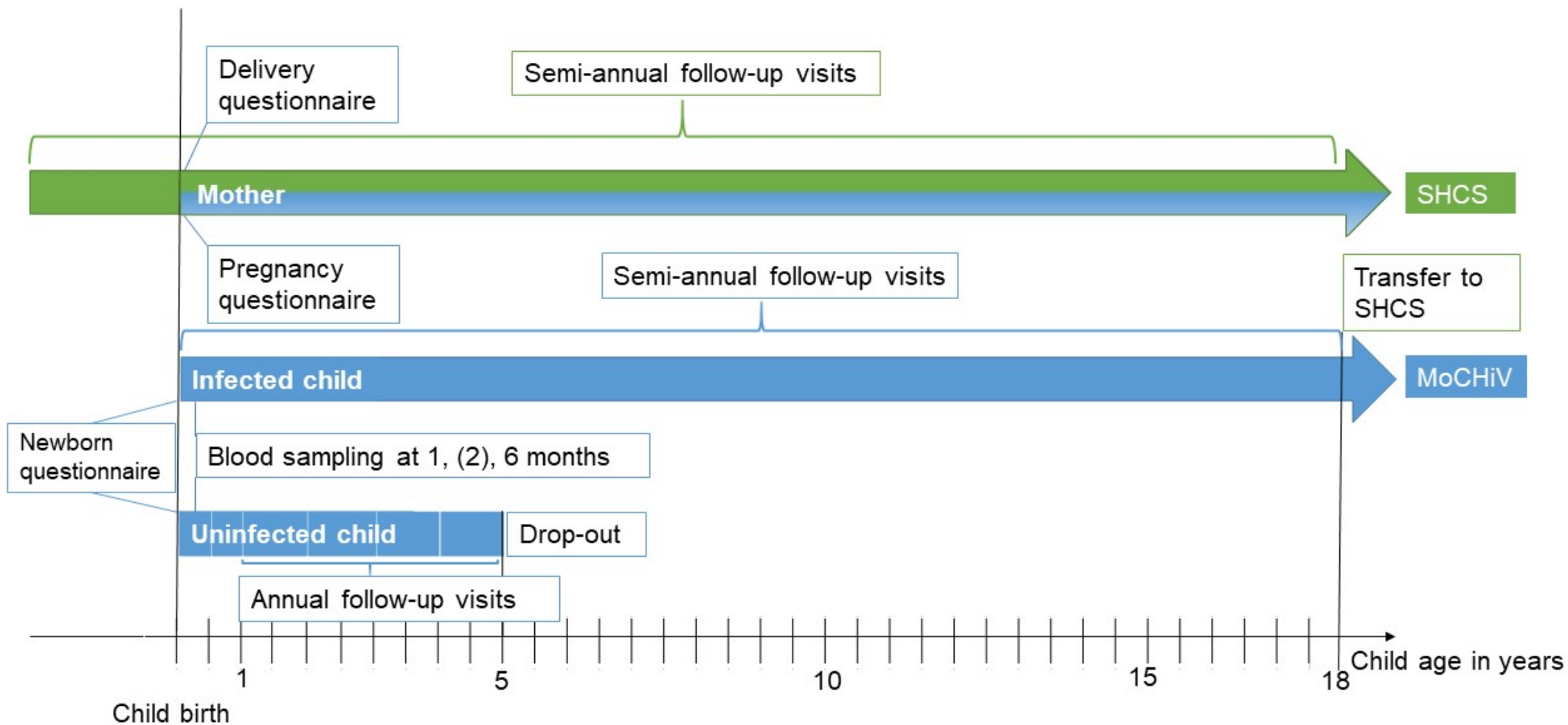

Supplement: online supplemental file 2 [file bmjopen-14-9-s002.pdf]
