# Supplementary material for: Cohort profile: the Swiss Mother and Child HIV Cohort Study (MoCHiV)
Source: BMJ Open. 2024 Sep 23;14(9):e086543. doi: 10.1136/bmjopen-2024-086543 (PMC11418562; doi:10.1136/bmjopen-2024-086543)
Supplement: Supplementary file 3 [file bmjopen-2024-086543.reviewer_comments.pdf]

PEER REVIEW HISTORY

BMJ Open publishes all reviews undertaken for accepted manuscripts. Reviewers are asked to complete a checklist review form and are provided with free text boxes to elaborate on their assessment. These free text comments are reproduced below.

ARTICLE DETAILS

**TITLE (PROVISIONAL)**	Cohort profile: The Swiss Mother and Child HIV Cohort Study (MoCHiV)
**AUTHORS**	Paioni, Paolo; Capaul, Murezi; Brunner, Anja; Traytel, Anna; Aebi-Popp, Karoline; Crisinel, Pierre-Alex; Duppenthaler, Andrea; Günthard, Huldrych; Martinez De Tejada, Begona; Kottanattu, Lisa; Stockle, Marcel; Rauch, Andri; Wagner, Noemie; Hoesli, Irene; Rudin, Christoph; Scherrer, Alexandra; Kusejko, Katharina; Kahlert, Christian

VERSION 1 - REVIEW

**REVIEWER NAME**	Goetghebuer, Tessa
**REVIEWER AFFILIATION**	CHU St Pierre, Pediatrics
**REVIEWER CONFLICT OF INTEREST**	No COI
**DATE REVIEW RETURNED**	17-Apr-2024

**GENERAL COMMENTS**	This paper summerizes the data collected in Swiss cohorts of children living with HIV (CWH) and births from HIV-infected mothers from 1986 to 2022. It also raises major contributions made by the authors on management of CWH and HEU. It is well and clearly writen, tables and figures are nice. A few minor clarification would be welcome: The number of mothers registered and described in Table 1 is 1041 and the number of children is 2154. It implies that some women had more than 1 child and it is probable also that some twin deliveries occured. This information could be added in the table or in the text. Still in Table 1, if the age of the mother at birth of the first child is mentionned, it is not specified wether it is the case for VL at delivery or if all deliveries have been considered. if it is the case, I would suggest to transfer the information in the same table but under the children characteristics together with mode of delivery.<br>In the text, I would suggest to separate the paragraph about CWH (line 273-284) from the rest of the text that relates to PMTCT. In this paragraph, implementation of early treatment of HIV-infected infants should be described as it represents an important step in the management of CWH. It would be also interesting the describe the processus of transition to adult care and the age it happens, in link with Table 2.

**REVIEWER NAME**	Van de Perre, Philippe
**REVIEWER AFFILIATION**	INSERM UMR 1058, Pathogenesis and control of chronic infections
**REVIEWER CONFLICT OF INTEREST**	I have no competing interest with this work.
**DATE REVIEW RETURNED**	15-May-2024

GENERAL COMMENTS	Major comments<br><br>- In the Abstract section, paragraph on future plans (lines 72-74): In addition to investigations on other vertically transmitted pathogens, it would be nice to have a clear prospective view on the health problems encountered in HIV-exposed uninfected children (HEU) and their pathogenic origin.<br>- Throughout the document, percentages should be given as numerator/denominator (%, with 95% confidence interval).<br>- Lines 287-290: "In 2016, Switzerland became the first country in the world to recommend the discontinuation of nPEP in newborns born to WWH with fully suppressed HIV pVL at the time of delivery, regardless of the mode of delivery": How many countries (excluding Switzerland) have adopted this recommendation? According to Figure 2, the number of newborns receiving nPEP had already decreased before the Swiss recommendation was issued. What is the explanation for this?<br>- Line 295: The new 2018 guidelines on breastfeeding are mentioned, but the content of these guidelines is not detailed.<br>- Line 305: at the end of the paragraph on postpartum follow-up, it would be interesting to have the authors' opinion on the possibility of revising the breastfeeding guidelines in the light of the results of recent studies (e.g. Kankasa et al, Lancet 2024), at least in women with a detectable viral load in the postpartum period.<br>- Paragraph on collaboration and active patient involvement, line 358: it would be interesting to know what use was made of the cohort's biobanks (collections of biological samples): what process for sample transfer? frequency of requests? number of samples transferred to external research teams? type of studies?<br>- It would be very informative to have a box or table summarising, in chronological order, the Swiss recommendations on the prevention of vertical transmission of HIV and on infant feeding.<br><br>Minor comments<br><br>- Lines 209-210: please use the term "high-income countries" instead of "industrialized countries".<br>- Lines 220-223: please change "Pediatric AIDS Clinical Trials Group Protocol 076 Study Group..." to "Pediatric AIDS Clinical Trials Group Protocol 076 (PACTG-076) Study Group".<br>- Line 228: What does "additive" protective effect mean? Additional? Cumulative?

VERSION 1 – AUTHOR RESPONSE

Reviewer: 1

Tessa Goetghebuer, Saint-Pierre University Hospital Department of Paediatrics

Comments to the Author:

This paper summarizes the data collected in Swiss cohorts of children living with HIV (CWH) and births from HIV-infected mothers from 1986 to 2022. It also raises major contributions made by the authors on management of CWH and HEU. It is well and clearly written, tables and figures are nice. A few minor clarification would be welcome:

The number of mothers registered and described in Table 1 is 1041 and the number of children is 2154. It implies that some women had more than 1 child and it is probable also that some twin deliveries occurred. This information could be added in the table or in the text.

In fact, a total of 1446 children were born from 1041 WWH. This reflects to:

- 706 mothers with 1 child registered
- 280 mothers with 2 children registered
- 42 mothers with 3 children registered
- 11 mothers with 4 children registered
- 2 mothers with 5 children registered

Of those children, we have 25 twins and 1 triplet registered. The rest (i.e. diVerence from 2154 to 1446) reflects children where the mother was not enrolled in the SHCS. This information was added to the manuscript under the section “Numbers of pregnant women with HIV and vertical transmission” and now reads as follows.

A total of 1446 children were born from the 1041 WWH mentioned above, with 335 mothers having more than one child registered (280 mothers having 2, 42 mothers having 3, 11 mothers having 4 and 2 mothers having 5 children registered). In total, 25 twins and one triplet were born.

Still in Table 1, if the age of the mother at birth of the first child is mentioned, it is not specified whether it is the case for VL at delivery or if all deliveries have been considered. if it is the case, I would suggest to transfer the information in the same table but under the children characteristics together with mode of delivery.

4

Thank you for this important observation. In fact, viral load in Table 1 referred to viral load at delivery of the first child. Table 1 was modified to represent viral load at delivery for all deliveries (i.e. including mothers with multiple deliveries).

In the text, I would suggest to separate the paragraph about CWH (line 273-284) from the rest of the text that relates to PMTCT.

The information about CWH was moved to the end of the “Findings to date” section.

In this paragraph, implementation of early treatment of HIV-infected infants should be

described as it represents an important step in the management of CWH.

We agree with the reviewer that implementation of early treatment of HIV-infected infants has been an important step in the management of CWH. Nevertheless, in this section we describe the most relevant findings from our cohort study and since no publications on this topic were generated using cohort data we decided not to include implementation of early treatment in this section.

It would be also interesting the describe the processus of transition to adult care and the age it happens, in link with Table 2.

We agree with the reviewer, that it would be interesting to describe the transition process, but at present standardized data regarding transition to adult care are not collected in our cohort. Additionally, the transition process is individually shaped at each centre in Switzerland. A common national recommendation for transition to adult care is lacking as it is also the case in international guidelines on the management of paediatric HIV as the EACS/Penta guidelines for example. We added following information in the section “Numbers od HIV exposed children and children with HIV”:

The median age at the last visit in MoCHiV before transfer to the SHCS was 18.0 years (IQR 16.5 - 18.8).

Reviewer: 2

Prof. Philippe Van de Perre, INSERM UMR 1058, Université de Montpellier

Comments to the Author:

This manuscript summarises the description, organisation and main results of the pioneering Swiss paediatric HIV cohort study (The Swiss Mother and Child HIV Cohort Study - MoCHiV) initiated in January 1986. Although quite descriptive and extensive, the manuscript is clear and easy to read.

Major comments

- In the Abstract section, paragraph on future plans (lines 72-74): In addition to investigations on other vertically transmitted pathogens, it would be nice to have a clear prospective view on the health problems encountered in HIV-exposed uninfected children (HEU) and their pathogenic origin.

Thank you for pointing this out. We have added more perspectives to the paragraph. It now reads as follows.

Future plans: Most children present within the cohort are now HEU, highlighting the need to investigate other vertically transmitted pathogens such as hepatitis B and C viruses, cytomegalovirus or Treponema pallidum. In addition, long-term health outcomes (e.g. the accumulation of resistance mutations) in young adults who acquired HIV vertically will be compared to young adults who acquired HIV horizontally.

- Throughout the document, percentages should be given as numerator/denominator (%, with 95% confidence interval).

We adapted the manuscript where necessary to always give numerator and denominator for percentages. We decided not to calculate 95% confidence interval for percentages because all reported values describe the actual numbers reported within MoCHiV, and not estimations. Adding confidence intervals would only be possible when assuming that this study is randomly sampled from a larger population – but since the MoCHiV study might include a biased subsample of all pregnant WWH and CWH in Switzerland, also with the actual total numbers being unknown over time, we think that adding CIs would rather lead to more confusion and decrease readability of the respective paragraphs.

- Lines 287-290: "In 2016, Switzerland became the first country in the world to recommend the discontinuation of nPEP in newborns born to WWH with fully suppressed HIV pVL at the time of delivery, regardless of the mode of delivery": How many countries (excluding Switzerland) have adopted this recommendation?

We are not aware of any other countries that have oVicially discontinued the nPEP to date. There was a recent survey in 20 countries from the WHO European Region by EPPICC/PENTA (https://penta-id.org/hiv/eppicc/) in which participants from three other European countries self-reported that they had discontinued nPEP in specified situations in some parts of their country. As the results of this survey are currently under review, please understand that they cannot be further specified here.

According to Figure 2, the number of newborns receiving nPEP had already decreased

before the Swiss recommendation was issued. What is the explanation for this?

In Figure 2 the absolute number of newborns receiving nPEP decreased from 2014 to 2015 because of the decrease in the total number of deliveries, but the proportion of children receiving nPEP remained stable as in previous years. It was only in 2016 with the change in the national recommendations that the proportion dropped dramatically.

- Line 295: The new 2018 guidelines on breastfeeding are mentioned, but the content of these guidelines is not detailed.

To further clarify the content, we have amended the paragraph. It now reads as follows. We would like to point out that we also cite a publication which, in addition to the

detailed content of the new recommendations, lists all the arguments in favour of the new approach.

In 2018, new guidelines on breastfeeding in WWH were published. This made Switzerland the first country in a resource-rich setting to no longer rigidly discourage breastfeeding in WWH, provided certain defined conditions are met and monitoring is ensured (32). These conditions are referred to as the “optimal scenario” and include a fully suppressed maternal HIV viral load throughout pregnancy. If the optimal scenario conditions are met, breastfeeding is supported if the mother wishes to breastfeed. However, the decision is preceded by a shared decision-making process to ensure that the pregnant woman fully understands the risks and benefits of breastfeeding in HIV. Thus, there is currently no recommendation of breastfeeding with HIV in Switzerland.

- Line 305: at the end of the paragraph on postpartum follow-up, it would be interesting to have the authors' opinion on the possibility of revising the breastfeeding guidelines in the light of the results of recent studies (e.g. Kankasa et al, Lancet 2024), at least in women with a detectable viral load in the postpartum period.

Thank you for this comment. In the current Swiss recommendations, a detectable maternal HIV pVL is not compatible with further breastfeeding support. This means that the confirmed detection of a maternal HIV viral load after birth always leads to the cessation of breastfeeding.

Nevertheless, it has been decided to revise the recommendations. The question of

initiating treatment of the infant if a viral load is detected certainly needs to be reassessed. Fortunately, the detection of a maternal viral load after delivery is an absolute exception in Switzerland.

- Paragraph on collaboration and active patient involvement, line 358: it would be interesting to know what use was made of the cohort's biobanks (collections of biological samples): what process for sample transfer? frequency of requests? number of samples transferred to external research teams? type of studies?

The section Collaboration and active patient involvement was amended as follow to include information on the use of the cohort’s biobank. Information about the cumulative number of transferred samples and the frequency of requests are not available in the cohort database as each request is evaluated by the scientific board and, if accepted, processed on an individual base.

As described in the findings to date and strengths of the cohort, collaboration with other cohorts such as the European Pregnancy and Paediatric Infections Cohort Collaboration (EPPICC) is already in place and is considered important. In principle, any access to cohort data or samples to answer a research question, whether internal or external, or the request for collaboration requires the approval of the Scientific Board. For this purpose, a structured and detailed study proposal must be submitted. Biological samples were used for example to assess the rate of vertical transmission of antiretroviral drug-resistant HIV strains in CWH (42). Further information regarding the cohort biobank can be found in previous publications (1, 2). The template, submission deadlines and a description of the evaluation and decision-making process can be found on the website (http://www.shcs.ch/132-who-can-submit). Patient

representatives have been on the Scientific Board for several years, ensuring active patient involvement in research.

- It would be very informative to have a box or table summarising, in chronological order, the Swiss recommendations on the prevention of vertical transmission of HIV and on infant feeding.

This information is already summarized in chronological order in Figure 2 (graphically

with vertical dashed lines and explained in the legend).

Minor comments

- Lines 209-210: please use the term "high-income countries" instead of "industrialized countries".

The manuscript was adapted accordingly.

- Lines 220-223: please change "Pediatric AIDS Clinical Trials Group Protocol 076 Study Group..." to "Pediatric AIDS Clinical Trials Group Protocol 076 (PACTG-076) Study Group".

The manuscript was adapted accordingly.

- Line 228: What does "additive" protective eVect mean? Additional? Cumulative?

The term "additive" was changed to "cumulative".

VERSION 2 – REVIEW

**REVIEWER NAME**	Goetghebuer, Tessa
**REVIEWER AFFILIATION**	CHU St Pierre, Pediatrics
**REVIEWER CONFLICT OF INTEREST**	No competing interests to declare
**DATE REVIEW RETURNED**	13-Aug-2024

**GENERAL COMMENTS**	All the questions have been adequately adressed

**REVIEWER NAME**	Van de Perre, Philippe
**REVIEWER AFFILIATION**	INSERM UMR 1058, Pathogenesis and control of chronic infections
**REVIEWER CONFLICT OF INTEREST**	I have no competing interest.
**DATE REVIEW RETURNED**	13-Aug-2024

**GENERAL COMMENTS**	The authors have adequately addressed all suggested changes in this revised version, and I have no additional comments. The authors and teams should be commended for their pioneering work in the field of HIV PMTCT.
